# Asymmetric Facial Bone Fragmentation Mirrors Asymmetric Distribution of Cranial Neuromasts in Blind Mexican Cavefish

**DOI:** 10.3390/sym8110118

**Published:** 2016-10-31

**Authors:** Joshua B. Gross, Andrew Gangidine, Amanda K. Powers

**Affiliations:** 1 Department of Biological Sciences, University of Cincinnati, Cincinnati, OH 45221, USA; 2 Department of Geology, University of Cincinnati, Cincinnati, OH 45221, USA

**Keywords:** *Astyanax mexicanus*, lateral line system, laterality, left-right asymmetry

## Abstract

Craniofacial asymmetry is a convergent trait widely distributed across animals that colonize the extreme cave environment. Although craniofacial asymmetry can be discerned easily, other complex phenotypes (such as sensory organ position and numerical variation) are challenging to score and compare. Certain bones of the craniofacial complex demonstrate substantial asymmetry, and co-localize to regions harboring dramatically expanded numbers of mechanosensory neuromasts. To determine if a relationship exists between this expansion and bone fragmentation in cavefish, we developed a quantitative measure of positional symmetry across the left-right axis. We found that three different cave-dwelling populations were significantly more asymmetric compared to surface-dwelling fish. Moreover, cave populations did not differ in the degree of neuromast asymmetry. This work establishes a method for quantifying symmetry of a complex phenotype, and demonstrates that facial bone fragmentation mirrors the asymmetric distribution of neuromasts in different cavefish populations. Further developmental studies will provide a clearer picture of the developmental and cellular changes that accompany this extreme phenotype, and help illuminate the genetic basis for facial asymmetry in vertebrates.

## 1. Introduction

**Figure 1:**
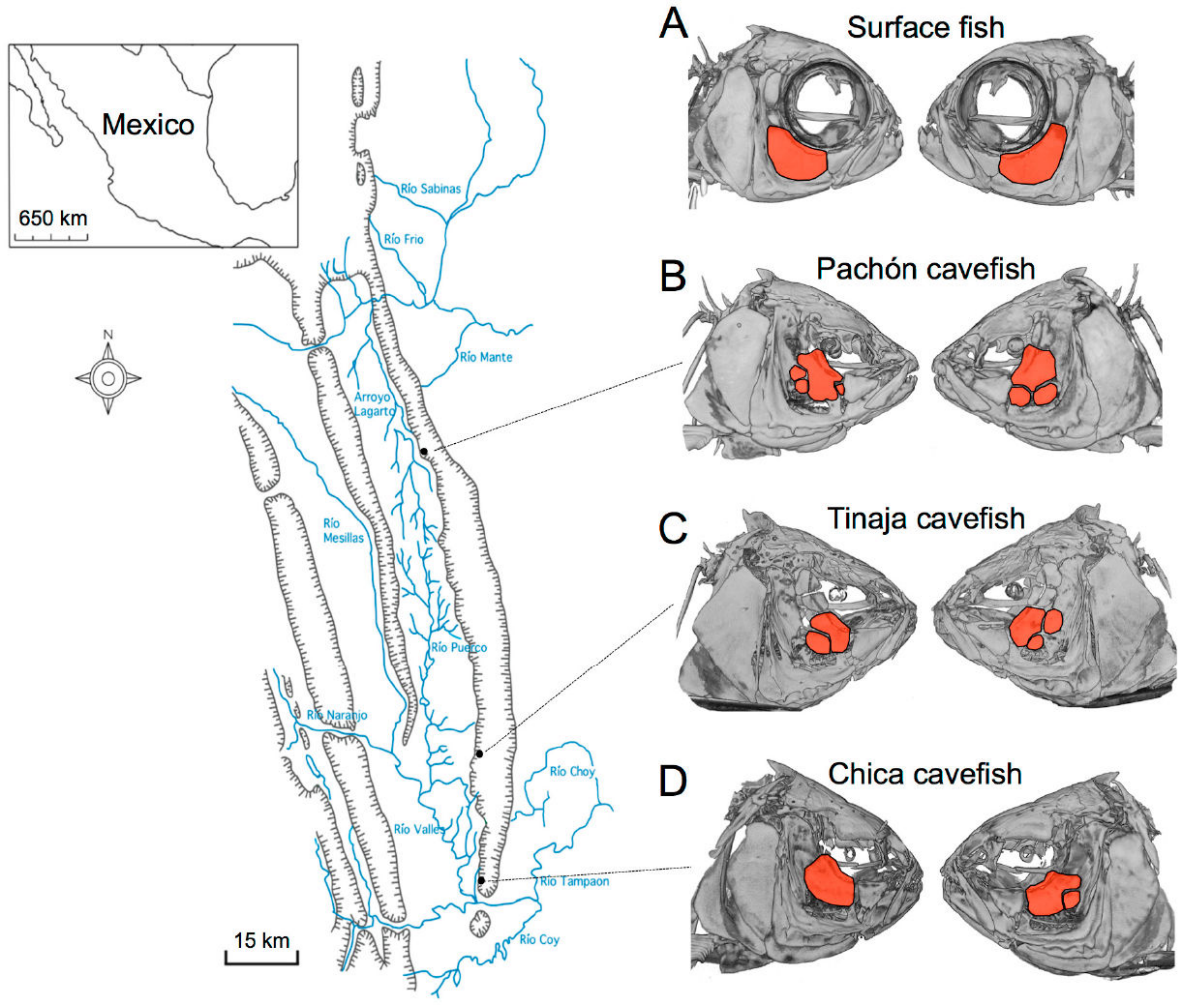
Fragmentation of the third suborbital bone (“SO3”) is present across the Sierra de El Abra landscape of blind Mexican cavefish populations. Surface-dwelling *Astyanax mexicanus* fish harbor largely symmetric cranial complexes, evidenced by symmetric shapes and sizes of SO3 bones (red, **A**); In contrast, fragmented SO3 bones (red, **B**–**D**) are present in representative individuals drawn from the Pachón (**B**), Tinaja (**C**) and Chica (**D**) cave localities. Note the bilateral asymmetric patterns of the number of fragmented bony elements present in cave-dwelling fish (**B**–**D**).

One of the first morphological aberrations described in blind Mexican cavefish, following their discovery in 1936, were facial bone fragmentations. Hubbs and Innes (1936) supposed that these fragments might have arisen from injury, perhaps during life or following specimen preservation [1]. However, subsequent discoveries of additional cavefish populations across the Sierra de El Abra landscape of northeastern Mexico demonstrated that fragmentation arises as a normative component of cavefish cranial bone development [2,3]. These fragmentation phenotypes have evolved in different cave populations (Figure 1), reviewed in [4].

The genetic and molecular basis for facial bone fragmentation, and its potential adaptive significance, remains entirely unknown. However, the principal bone that undergoes fragmentation in blind Mexican cavefish, the third suborbital bone (“SO3”), is densely populated by cranial sensory neuromasts of the lateral line system [5]. These mechanoreceptive structures are made up of sensory hair cells and are crucial signaling centers conferring a keen sense of water movements (e.g., hydrodynamic flow, vibration) and facilitating foraging in the total darkness of the cave [6]. Notably, surface-dwelling fish from the rivers and streams surrounding the El Abra cave complex never demonstrate the fragmentation phenotype [4]. Moreover, the density of cranial neuromasts overlying the SO3 bone in surface fish is modest in comparison to cavefish.

Recent studies have demonstrated that patterns of fragmentation in cavefish are asymmetric [7]. Further, analyses in zebrafish reveal that the facial bone morphology is associated with distribution patterns of cranial neuromasts [8]. In this report, we sought to establish a relationship between the pattern(s) of bone fragmentation and cranial neuromasts. An important challenge was the fact that cranial neuromast patterns (genetically, numerically and spatially) are highly complex. Therefore, we aimed to generate an index of symmetry between the left and right sides of individuals, in a quantitative manner.

**Figure 2:**
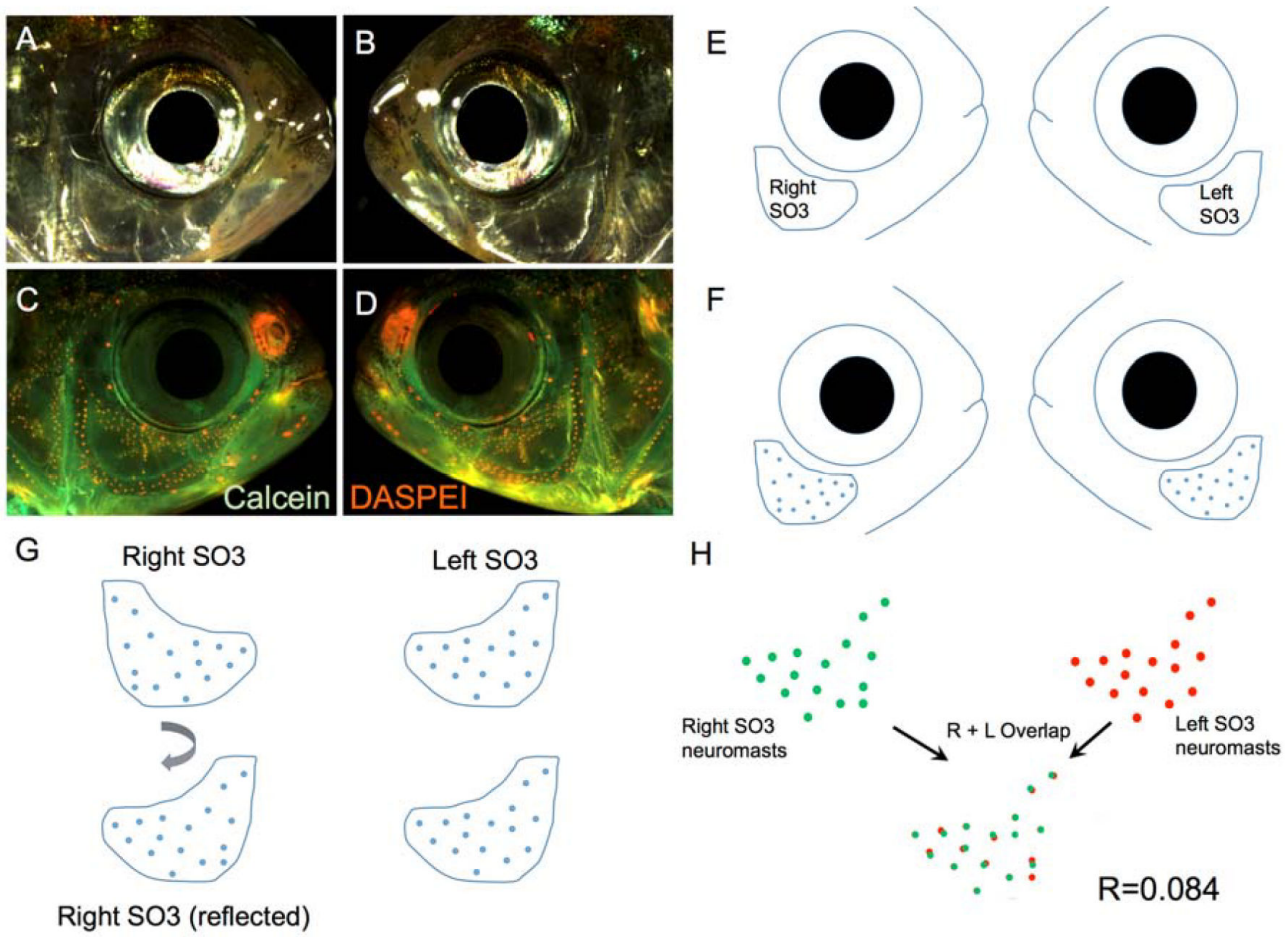
A method for quantifying symmetry for a complex phenotype. High-resolution light images are taken for the right (**A**) and left (**B**) sides of the face of a specimen. The live fluorescent dyes, Calcein (**green**) and DASPEI (**orange**), are implemented to visualize bone and neuromasts (**C**,**D**), respectively; A digital trace is made of the **left** and **right** sides of each individual (**E**), including an outline of the SO3 bone and neuromasts (**F**); One side of the digital representation is then reflected horizontally (**G**) to create a consistent orientation; Finally, the right- and left-sided distributions are quantitatively measured for `overlap' using the JACoP software tool (ImageJ; Methods) and a correlative value (*R*) is generated for each individual (**H**). The correlative value for this surface fish individual is *R* = 0.084.

Towards this end, we developed a method in which the precise positions of neuromasts were digitally defined and scored independently on the left and right sides of the cranium. Once defined, digital representations of neuromast positions were reflected on one side of the face, and aligned to the contralateral side (Figure 2). Using a software program workflow, we calculated the degree of overlap (i.e., the correlation) in cranial neuromast position between the left and right facial sides. By performing this analysis in multiple individuals, we were able to calculate an average “measure” of symmetry across representative members of three different cavefish populations and the surface-dwelling fish population.

Bone morphology impacts neuromast position in other fish species [8]. Therefore, we reasoned that superficial neuromast pattern and positioning would demonstrate higher measures of symmetry in surface versus cave-dwelling populations. This follows the observation that surface fish demonstrate near perfect cranial symmetry with respect to the facial bones that encircle the eye (the “circumorbital” series) [4]. In contrast, cave-dwelling populations harbor substantial asymmetry across the left-right axis for this bony complex at both the individual and population levels [4,7].

We discovered that three different cavefish populations were less symmetric compared to the surface-dwelling fish population. Interestingly, the degree of asymmetry does not appear to significantly differ between cavefish populations, despite the fact that each cave entrance is geographically distant from one another [9,10]. Moreover, we discovered that within cavefish populations, the degree of bilateral neuromast asymmetry correlates to the degree of left-right bony asymmetry across individuals. These results conform to our expectations, and imply that asymmetry at the level of cranial bones reflects asymmetry in the pattern of cranial neuromasts. At present, the polarity of this association remains unknown. In principle, altered bony morphology may disrupt neuromast positioning. Alternatively, aberrant neuromast positioning may interfere with normal patterning and ossification of the facial bones. Future developmental studies evaluating the ontogeny of both processes simultaneously will provide further insight to the nature of this relationship.

This work provides the first quantitative metric for measurement and comparison of cranial neuromast position and patterning asymmetry. This tool will enable further genetic and developmental studies to gain insight to the underpinnings of asymmetry in natural cavefish populations. This work, in turn, will shed light on the broader forces leading to cranial asymmetry in vertebrates, a poorly understood phenomenon. In the context of cavefish evolution, this work will also allow us to understand if asymmetry evolves through neutral forces (e.g., genetic drift) or if strong selection for one trait (increased lateral line sensitivity) indirectly impacts a seemingly unrelated morphological trait (bone morphology).

## 2. Materials and Methods

### 2.1. Animal Rearing

All fish were maintained under identical rearing conditions in a satellite fish husbandry room at the University of Cincinnati. Fish were housed under a 12 h light:12 h dark lighting schedule and fed dry flake food (TetraMin Pro) once daily. All individuals used in this study were maintained in a husbandry unit (Aquaneering, San Diego, CA, USA) receiving reverse-osmosis water treated to achieve a pH of 7.4 (±0.2) and conductivity of 800 μS (±50 μS). Each fish tank received separate water delivery and drainage; and a recirculating pump provided filtration through coarse and fine mechanical filters, a biofilter, a micron filter, and a UV filter. Individuals were either housed in groups (five or 10 gallon glass tanks) or individually in 1 L BPA-free plastic tanks.

### 2.2. Live Staining and Imaging

Thirty adult individuals were selected from our stock of surface fish, as well as 30 adult individuals from three different cave localities (Pachón, Tinaja, and Chica). Each individual was stained with Calcein (Sigma Aldrich; St. Louis, MO, USA) and 2-[4-(Dimethylamino)styryl]-1-ethylpyridinium iodide (DASPEI; Sigma Aldrich) in order to visualize bone and neuromasts, respectively. The left and right side of each fish was imaged simultaneously to visualize fluorescent bone and neuromast co-labels. A montage was created for each image by stacking a series of Z-plane images at multiple focal points. This resulted in all neuromast organs and bone margins to remain focused, despite slight variation in the Z-plane. All micrographs were collected using a Leica M205FA stereomicroscope (Wetzlar, Germany) equipped with a DFC310FX color camera. Montage images were collected utilizing the MultiFocus module within the Leica Application Suite (LAS) software package (version 3.8, Leica Microsystems, Buffalo Grove, IL, USA, 2015).

### 2.3. Digital Analysis of Neuromast Position and Overlap

The SO3 bone (and any present SO3 fragmentation) and the “Crescent Region” (area demarcated by large canal neuromasts present beneath the eye of the fish and extending across the entire face) were outlined on each individual using the “freehand” tracing tool in Microsoft Powerpoint (v14.3.5, Microsoft, Redmond, WA, USA, 2011). The area of each Crescent and SO3 region (including individual fragmentations of SO3 bones) was obtained in pixels using the basic “measure” function on the image analysis software ImageJ (v2.0.0-rc-43/1.50e, ImageJ/NIH, Bethesda, Maryland, USA, 2015), and converted to millimeters squared (mm^2^). Neuromasts were then quantified using the “Point” tool in ImageJ in order to count the number of neuromasts present within the Crescent and SO3 areas of each respective locality.

Each montage image was processed in Adobe Photoshop (CS3, Adobe Systems Incorporated, San Jose, CA, USA, 2015) and each neuromast was marked in a new layer by placing a small circular dot directly over the neuromast center using the `pencil' tool. Once each side of an individual was marked, the right side was reflected (i.e., “flipped”) 180° across the horizontal axis. Using neuromast canals as place markers for orientation, a “best fit” was aligned for each right-left overlap. This was performed in technical triplicate to minimize investigator error. Once the “best fit” was decided, the background montage image for both the lateral sides was removed, leaving only the overlapping dots for the left and right side of the individual on a blank background. All digital files were loaded into ImageJ's JACoP Colocalization Plugin and scored for overlap. Each analysis yielded an *R*-value dependent upon how closely each image matched with the other. Three trials were averaged to generate a mean correlative score for each individual. *R*-values were recorded for each locality to be compared for neuromast symmetry.

### 2.4. Statistical Analyses

To assess for group differences in symmetry, we conducted a one-way analysis of variance (ANOVA) using locality (e.g., surface fish, Pachón cavefish) as a grouping variable and the mean symmetry score (*R*-values) as the dependent variable. Our omnibus *F*-test was significant, indicating significant differences in symmetry measure between groups (*F*(3,116) = 23.99, *P* << 0.001). Post hoc comparisons using Student's *t*-tests revealed a pattern of results wherein symmetry scores did not differ across all three cavefish populations, and all cavefish populations differed significantly from surface fish. All statistical analyses were performed using Microsoft Excel for Mac (v14.3.5, Microsoft, Redmond, WA, USA, 2011) and StatPlus:mac LE (Build 6.1.7.0/Core v6.1.60) (v5.9.80, Analystsoft, Walnut, CA, USA, 2015).

### 2.5. Ethical Approval Statement

This study was performed in accordance with the Guide for the Care and Use of Laboratory Animals of the U.S. National Institutes of Health. The Institutional Animal Care and Use Committee (IACUC) of the University of Cincinnati approved the Animal Use Protocol utilized for these studies (protocol number: 10-01-21-01; date of approval: 1 April 2016).

## 3. Results

### 3.1. Digital Analysis of Neuromast Position Enables a Quantitative Index of Symmetry for a Complex Phenotype

We implemented a method of digitally identifying the precise positions of superficial neuromasts overlying the SO3 bone in representative individuals drawn from each of the four *Astyanax* populations: surface-dwellers, Pachón cavefish, Tinaja cavefish and Chica cavefish (Figure 2). The high-resolution microscopy we employed allowed us to capture focal variation in the third-dimension (i.e., “montage” imaging). This technique, combined with robust DASPEI labeling of superficial neuromasts (Figure 2A–D), provided the ability to qualitatively compare the precise positions of DASPEI-positive neuromasts on the left and right sides of the head (Figure 2E,F). In order to capture a quantitative measure of symmetry, we utilized a software program (JACoP, “Just Another Co-localization Program”; v2.0.0-rc-43/1.50e, ImageJ/NIH, Bethesda, Maryland, USA, 2015) that provides a statistical measure of overlapping correlation. This software program could only be used for neuromast distributions oriented to the same side. To achieve this, we lateralized digital representations of one-side neuromast positions (Figure 2G). Once oriented in the same direction, the degree of “overlap” between the left and right SO3 neuromast positions could be quantitatively compared (Figure 2H). This method provided a reliable and robust method for comparing a complex phenotype (neuromast positions) between the lateral sides of multiple individuals.

### 3.2. Surface Fish Demonstrate Significantly Higher Measures of Neuromast Symmetry Compared to Cavefish

Surface-dwelling fish demonstrate higher morphological symmetry in their cranial complex compared to cavefish populations [4]. For instance, while cavefish are asymmetric with respect to numerous features of their circumorbital bone series, surface fish have not been shown to demonstrate abnormalities [4]. These include a variety of fusions of the six dermal bones comprising the circumorbital series, as well as substantial “fragmentation” of the third suborbital bone. This latter (extreme) phenotype has only been observed in cavefish populations [7].

Since the position of superficial neuromasts is influenced by the position(s) of bones [8], we reasoned that left-right measures of symmetry for superficial neuromasts would be higher for surface fish compared to cavefish populations. Unexpectedly, measures of quantitative symmetry in surface fish were rather modest. Using the JACoP freeware program, complete overlap of the left and right neuromasts populating the SO3 bone would result in a correlative statistic of 1.0. Across *n* = 30 individuals, the average measure of overlap between the left and right sides of surface fish was *R* = 0.082 (Table 1). To determine if group-level differences in symmetry existed in our dataset, we performed a one-way ANOVA using locality (e.g., surface fish, Pachón cavefish) as a grouping variable and mean symmetry scores (Table 1) as the dependent variable. Our *F*-test result was highly significant, demonstrating substantial differences in symmetry between surface and three cavefish populations (*F*(3,116) = 23.99, *P* << 0.001). *Post hoc* comparisons demonstrated that symmetry scores did not differ between cavefish populations; however, all cavefish populations differed from surface fish (*p* < 0.05).

**Figure 3:**
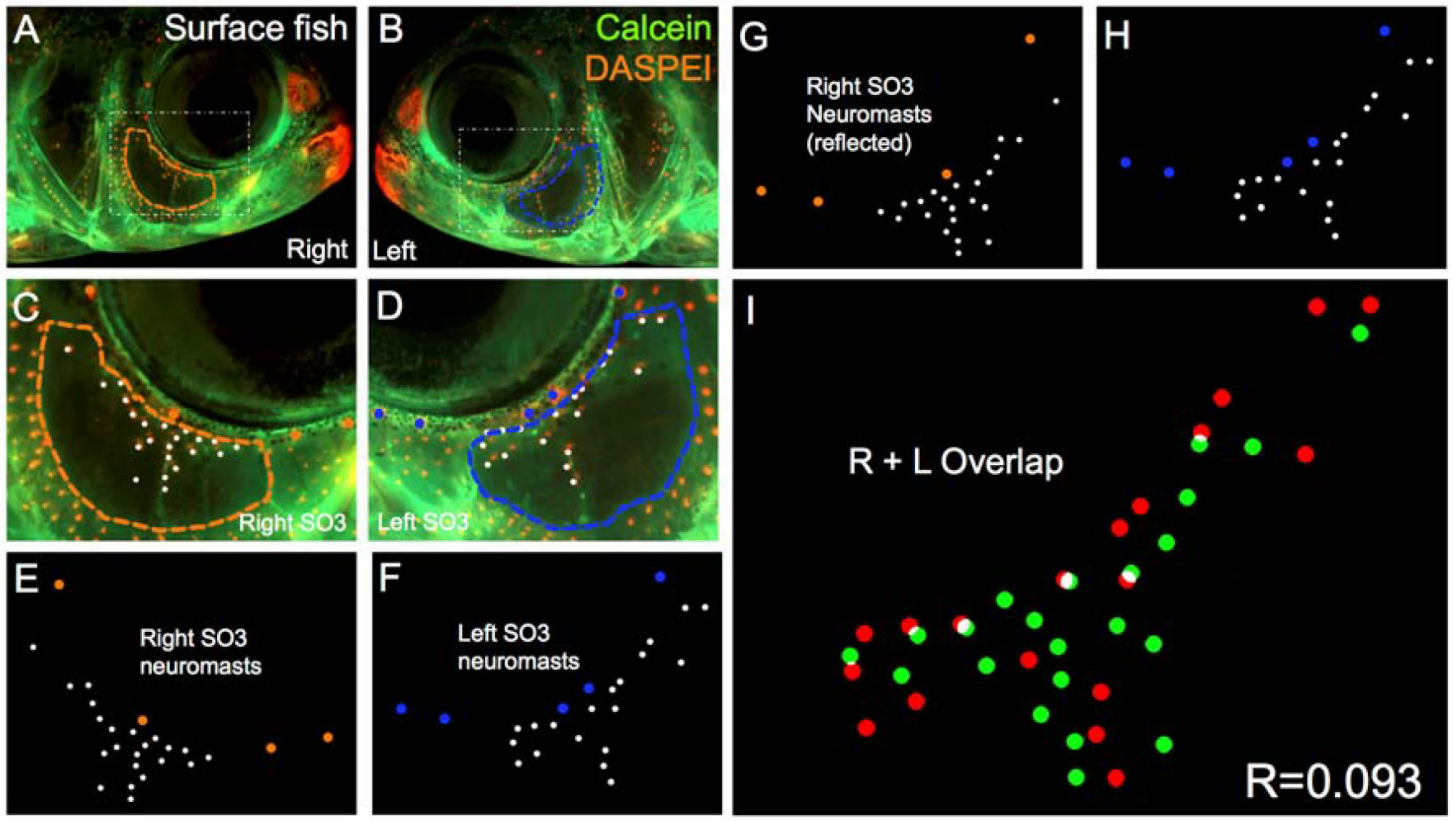
Surface-dwelling *Astyanax* fish demonstrate imperfect neuromast positional symmetry. Several surface morphs of *Astyanax mexicanus* were evaluated (*n* = 30) using live bone stain (**green**, **A**–**D**); and live neuromast stain (**orange**, **A**–**D**); The number of superficial neuromasts distributed across the SO3 bone were noted (**white** dots, **E**–**H**) relative to the positions of the right- (**orange** dots; **E**,**G**) and left-sided (**blue** dots; **F**,**H**) canal neuromasts; The right-sided SO3 neuromasts were reflected, allowing the right- and left-sided neuromast distributions to be compared quantitatively (**I**). The correlative value for this surface fish individual is *R* = 0.093.

This level of quantitative symmetry may reflect an “uncoupling” of neuromast position and patterning across the left-right axis, even in organisms demonstrating otherwise near-perfect cranial symmetry (Figure 3). Alternatively, the production and maintenance of neuromast organs in vivo may reflect a dynamic process of growth/division/branching [8]. We measured individuals at only one period in their life history—adulthood (>1 year). Therefore, in principle, even subtle differences in these processes across the left-right axis could manifest in the lower co-localization scores we measured in our putatively “symmetric” surface fish populations.

We sought, however, to quantify and compare the degree of symmetry in a natural population of fish. *Astyanax* fish are not considered to be a traditional model system that has been inbred for multiple generations. Moreover, some degree of facial asymmetry is normative [11]. Therefore, some asymmetry may be expected in our analyses as a consequence of the trait, our model organism, or both. Since the symmetry measure we developed for this study provides a continuous metric, we next sought to determine if comparisons of asymmetry between surface and cavefish conformed to our expectations.

### 3.3. Cavefish Individuals with Asymmetric Cranial Bones also Demonstrate Asymmetric Cranial Neuromasts

Given the results obtained for surface-dwelling fish, we next evaluated the positional symmetry of neuromast organs in three cavefish populations. Interestingly, we observed that fragmentation of the SO3 bone is a convergent trait. Specifically, SO3 fragmentation was found in all three cavefish populations despite substantial differences in their geographic positions (Figure 1). Much of the SO3 fragmentation we observed was asymmetric across the left-right axis, and therefore we assumed that asymmetric SO3 fragmentation would be associated with asymmetry of cranial neuromasts overlying this bone.

**Figure 4:**
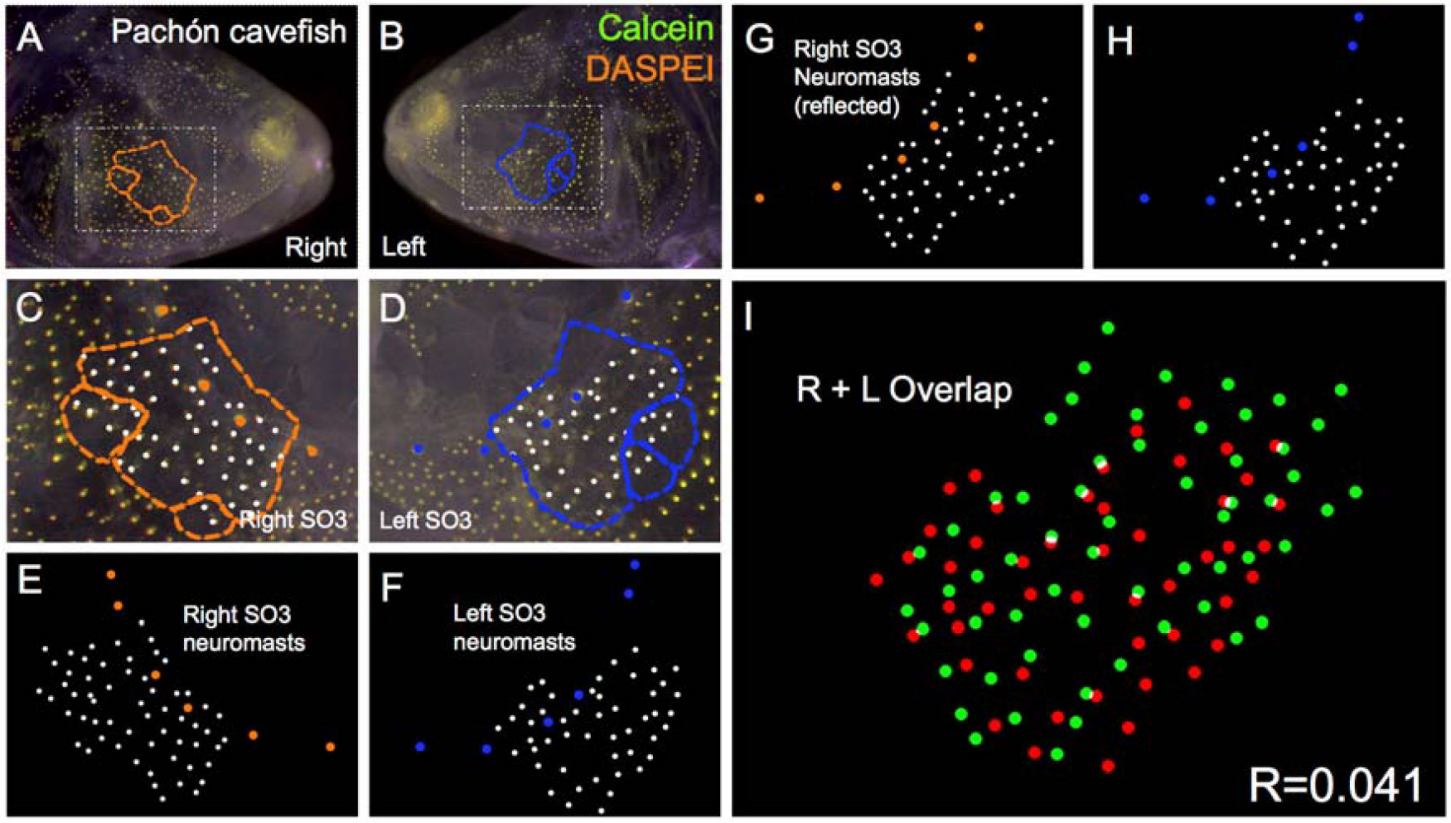
Pachón cave-dwelling *Astyanax* fish demonstrate asymmetric patterns of neuromast distributions. Several Pachón cave morphs of *Astyanax mexicanus* were evaluated (*n* = 30) using live bone stain (**green**, **A**–**D**); and live neuromast stain (**orange**, **A**–**D**); The number of superficial neuromasts distributed across the SO3 bone were noted (**white** dots, **C**–**H**) relative to the positions of the right- (**orange** dots; **C**,**E**,**G**); and left-sided (**blue** dots; **D**,**F**,**H**) canal neuromasts; The right-sided SO3 neuromasts were reflected, allowing the right- and left-sided neuromast distributions to be compared quantitatively (**I**). The correlative value for this Pachón cavefish individual is *R* = 0.041.

Pachón cavefish demonstrate some of the most extreme cave-associated phenotypes, including complete loss of eyes and melanic pigmentation (i.e., albinism). These fish are also isolated, residing in “perched” pools within limestone caves distributed across the El Abra region (Figure 1) [9]. Further, Pachón cavefish comprised the genetic mapping population in a recent genetic study documenting genetic and morphological asymmetry in the cranial complex [7]. We subjected *n* = 30 individuals to our asymmetry protocol and discovered consistently lower values of symmetry compared to surface-dwelling fish (Figure 4). The mean degree of symmetry across the left-right axis was *R* = 0.059 for Pachón cavefish (Table 1), compared to the surface fish mean degree of symmetry.

**Figure 5:**
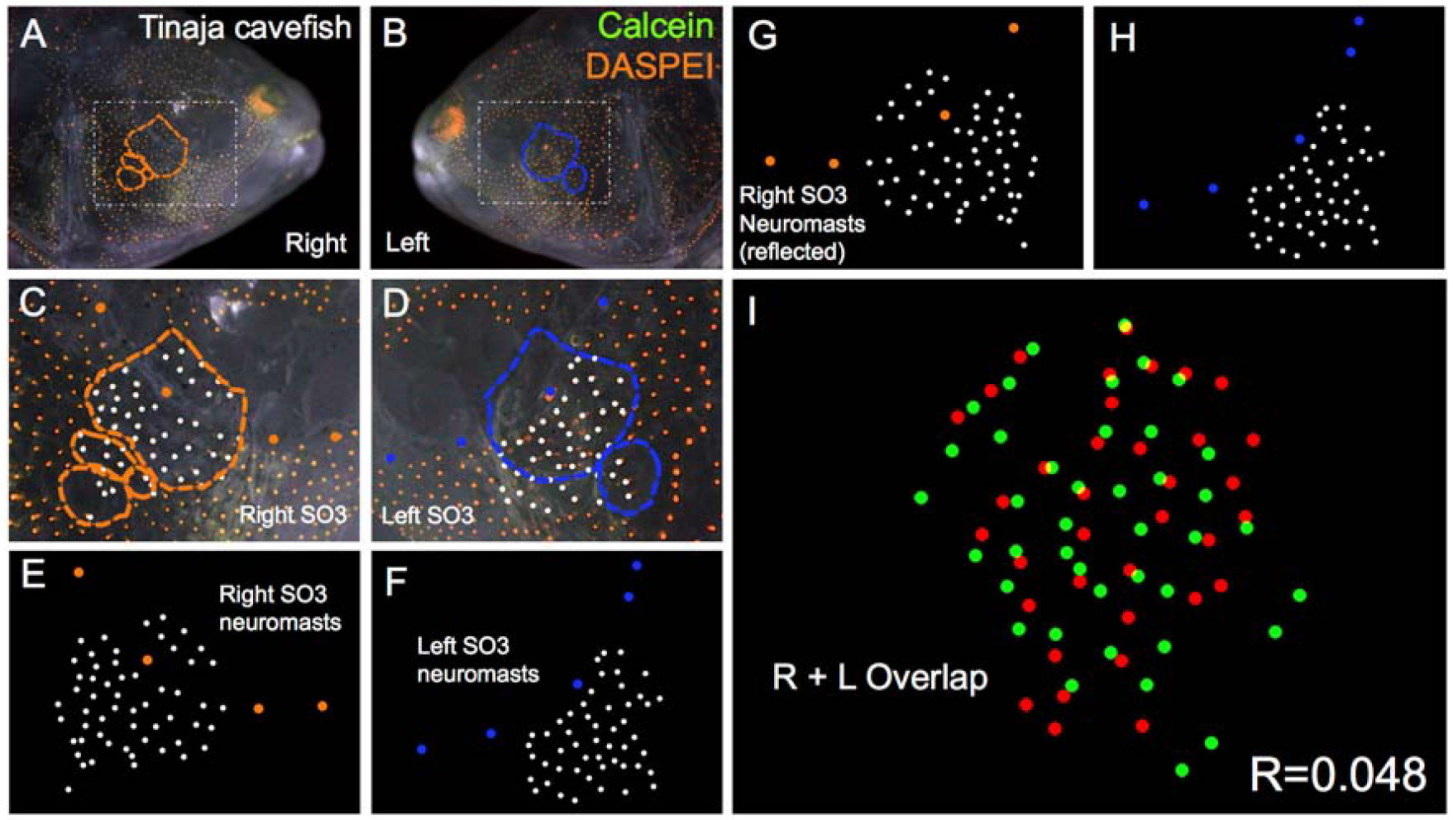
Tinaja cave-dwelling *Astyanax* fish demonstrate asymmetric patterns of neuromast distributions. Tinaja *Astyanax* cavefish were evaluated (*n* = 30) using live bone stain (**green**, **A**–**D**); and live neuromast stain (**orange**, **A**–**D**); The number of superficial neuromasts distributed across the SO3 bone were noted (**white** dots, **C**–**H**) relative to the positions of the right- (**orange** dots; **C**,**E**,**G**) and left-sided (**blue** dots; **D**,**F**,**H**) canal neuromasts; The right-sided SO3 neuromasts were reflected, allowing the right- and left-sided neuromast distributions to be compared quantitatively (**I**). The correlative value for this Tinaja cavefish individual is *R* = 0.048.

Tinaja cavefish, like Pachón cavefish, are regarded as an ancient population of cave-dwellers [10]. Although this population is part of the “El Abra” complex, it likely colonized the subterranean environment at a different time in the geologic past than Pachón [9]. Evidence to support this includes the fact that, unlike Pachón, Tinaja cavefish harbor some (albeit reduced) melanic pigmentation [12]. Further, although Tinaja cavefish demonstrate regressed vision, they lose their eyes at a slower pace compared to Pachón. Despite these subtle differences in morphological regression, both Tinaja and Pachón demonstrate SO3 bone fragmentation (Figure 1). Further, these patterns of SO3 fragmentation differ across the left-right axis. Interestingly, the qualitative nature of SO3 fragmentation (i.e., positioning of fragments) in Tinaja differs from Pachón (data not shown). When we measured the degree of asymmetry (Figure 5), we found that the average measure of symmetry was *R* = 0.054 (Table 1), significantly lower than that of the surface-dwelling fish. This symmetry score significantly differed from surface-dwelling fish; however, it did not differ significantly from the Pachón cave population.

**Figure 6:**
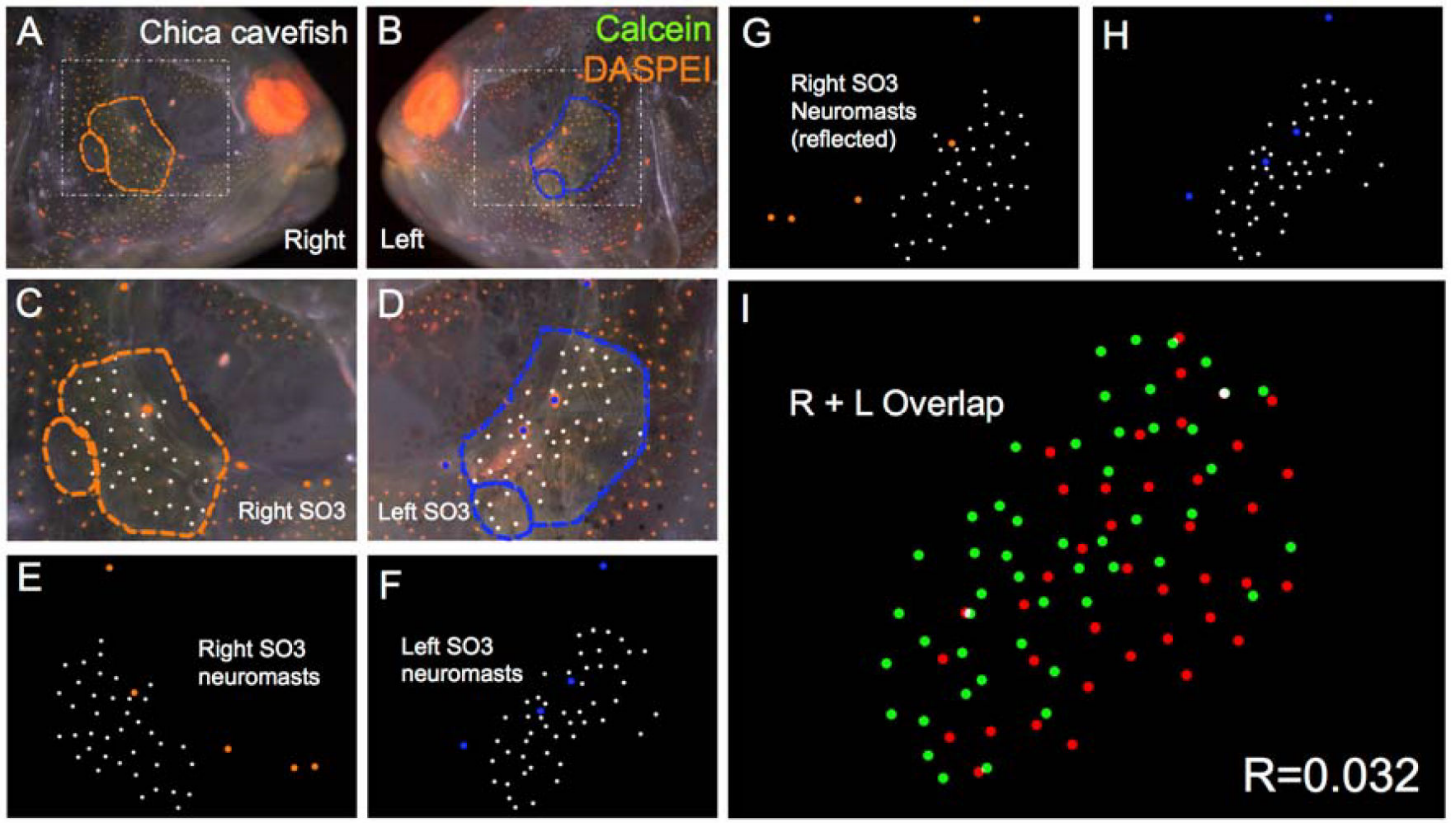
Chica *Astyanax* cavefish demonstrate asymmetric patterns of neuromast distributions. Several Chica cavefish of *Astyanax mexicanus* were evaluated (*n* = 30) using live bone stain (**green**, **A**–**D**); and live neuromast stain (**orange**, **A**–**D**); The number of surface neuromasts distributed across the SO3 bone were noted (**white** dots, **C**–**H**) relative to the positions of the right- (**orange** dots; **C**,**E**,**G**); and left-sided (**blue** dots; **D**,**F**,**H**) canal neuromasts; The right-sided SO3 neuromasts were reflected, allowing the right- and left-sided neuromast distributions to be compared quantitatively (**I**). The correlative value for this Chica cavefish individual is *R* = 0.032.

The last cavefish population we evaluated was a commercially derived cavefish population, most likely drawn from the Chica cave locality (Figure 6). This population was the first to be discovered in 1936, and has long been regarded as a “hybrid” population of surface fish and cavefish based on natural history, geographic and genetic studies [9,10]. These fish demonstrate regressed pigmentation and vision; however, the nature of these changes is not extreme. There is substantial phenotypic variability of these traits within the population, and this variability may be caused by periodic hybridization in the recent and distant past, with surface-dwelling fish from the surrounding region [9]. Interestingly, members of this population still demonstrate SO3 bone fragmentation, and this fragmentation appears to be asymmetric in nature (Figure 6). When we performed our left-right analyses of neuromast position, we found a significantly lower score *R* = 0.047 for Chica fish compared to surface fish (Table 1). Further, the scores for Chica, Pachón and Tinaja did not significantly differ from one another, suggesting that neuromast positional asymmetry is quite similar across different *Astyanax* cavefish localities.

## 4. Discussion

The distribution of cranial neuromasts on the head of fish and amphibians presents itself as a highly complex phenotype, likely subject to a variety of genetic and environmental influences [13,14]. Given this complexity, we sought to develop a tool that would enable us to compare the distributions of neuromasts on one side of the head against the other. We reasoned that neuromast positions are associated with the morphology of surrounding bony features. This association was established in *Danio rerio*, wherein the use of an *Edn1*-morpholino resulted in an altered morphology of the opercle bone and the neuromasts distributed on this bony element [8,15].

In this report, we present a technique for quantifying the degree of symmetry between left and right-sided cranial neuromasts in natural populations of cavefish. Based on prior work establishing the bone-neuromast relationship, we specifically tested if cranial bone asymmetry is associated with asymmetry in neuromast positioning. By combining live-stain imaging and high-resolution microscopy with digital analyses of form, we obtained a metric of “overlap” between neuromasts present on the left and right sides of the facial skeleton. Somewhat unexpectedly, we found that surface-dwelling fish demonstrate a low mean correlation between right- and left-sided neuromast positioning. From this result, we conclude that animals demonstrating generally symmetric cranial bone features still demonstrate some degree of “uncoupling” of neuromast organ positions between the left and right sides of the head. In principle, the mismatch could stem from subtle patterning, developmental, or environmental differences between the neuromasts populating the left and right sides of the head.

**Figure 7:**
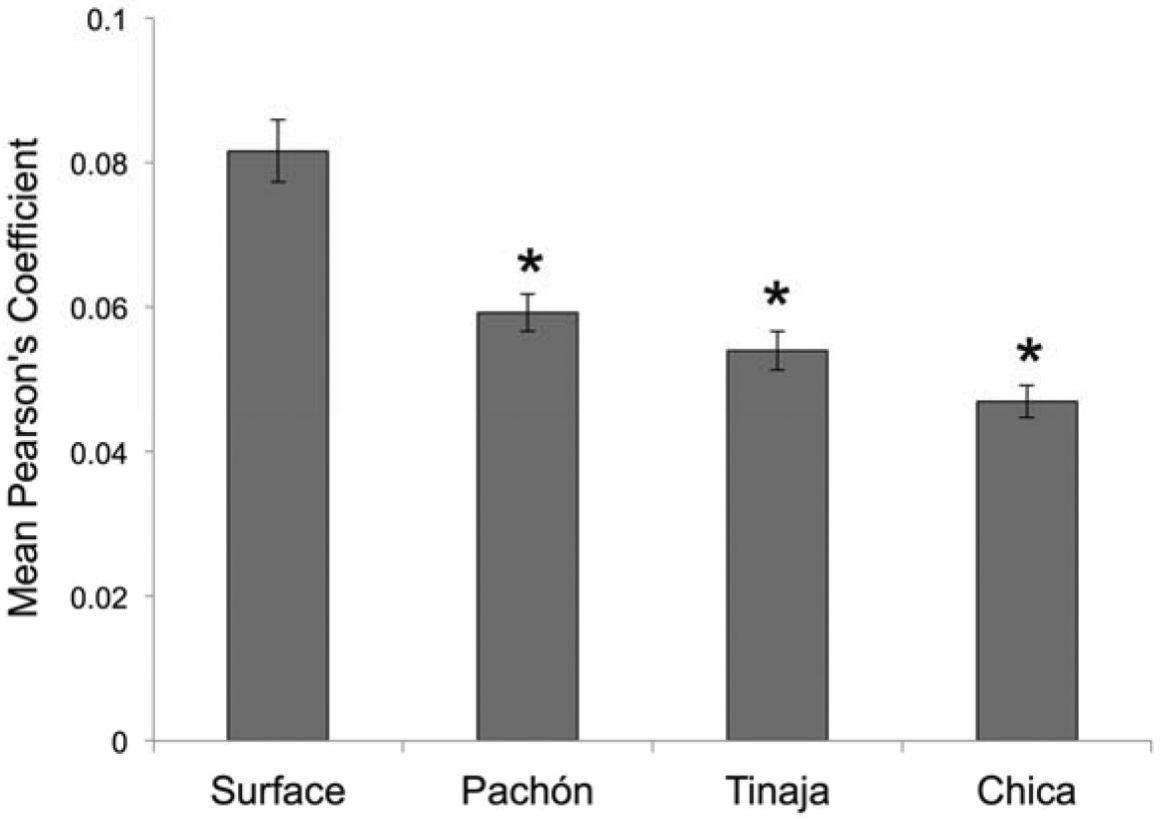
*Post hoc* statistical analyses reveal significant differences in quantitative symmetry between *Astyanax* fish. We evaluated if cave-dwelling forms demonstrate the same degree of asymmetry in neuromast patterning as surface-dwelling fish. Based on the results of a highly significant one-way Analysis of Variance (ANOVA; *p* << 0.001; see Section 2), we performed *post hoc* independent Student's *t*-tests and found that each of three cavefish populations harbored significantly lower values of mean neuromast positional symmetry compared to surface-dwelling fish. * *p* < 0.05; *n* = 30 individuals per group.

Our results demonstrated, however, that despite these left-right axial differences in surface fish, measures of symmetry were significantly lower for cave-dwelling fish. This study focused on three populations drawn from the “El Abra” complex of caves (Figure 1), which were colonized at different times in the geologic past [9]. Subsequent statistical analyses demonstrated that all cavefish populations uniformly differed from surface fish (Figure 7). However, no differences in average symmetry scores were observed between cave populations. Thus, SO3 bone fragmentation has evolved in these three cavefish populations alongside alterations of neuromast positioning.

It has long been appreciated that *Astyanax* cavefish harbor a far greater number of neuromasts compared to surface-dwelling fish [6]. Recent behavioral studies suggest that amplification of the lateral line sense confers sensitivity to water vibrations that facilitate foraging in the darkness of the cave [5,6]. In the dark and nutrient-poor microenvironment of the cave, this feature would presumably arise under very strong selection. The question of whether (and how) SO3 bone fragmentation is adaptive for *Astyanax* cavefish, however, remains unknown. Although this phenotype was observed in the holotype specimens used for species delimitation in 1936, it was mistakenly attributed to injury [1]. SO3 fragmentation was later discovered in several cave populations across the Sierra de El Abra, and by 1977, was believed to arise strictly as an indirect consequence of orbital collapse [16]. However, recent embryological and genetic analyses demonstrate that this trait harbors a genetic and developmental basis independent of visual system demise [17].

Thus, SO3 bone fragmentation is a novel and extreme phenotype present across several different *Astyanax* cavefish populations. The anatomical region overlying the SO3 bone is also a “hotspot” for numerical expansion of neuromasts in cave populations [5]. The neuromasts in this region of the facial skeleton are behaviorally linked to vibration attraction behavior (likely a strongly selected trait in cavefish) [5]. In this context, it is intriguing to consider that the two phenotypes may be developmentally and/or evolutionarily linked. The current, and previous, studies provide some support for the notion that bone fragmentation may evolve as a secondary consequence of selection for neuromast numerical expansion. However, not all cave populations demonstrate robust neuromast-mediated behaviors, and therefore future studies should focus on determining if bone fragmentation can evolve in the absence of neuromast expansion. If so, this may suggest a cryptic selective advantage to facial bone fragmentation evolving in the darkness of the cave environment. Irrespective, at present it is clear that the association between neuromast patterning and bone morphology persists in *Astyanax* cavefish. This system will continue to provide powerful insight to both the developmental and genetic bases of craniofacial asymmetry, and further illuminate how organisms adapt to extreme environments.

## Figures and Tables

**Table 1:** Mean symmetry scores for four *Astyanax* populations^1^.

Population	Mean Symmetry Score (*R*)	+/−SD	*N*
Surface fish	0.082	0.024	30
Pachón cavefish	0.059	0.014	30
Tinaja cavefish	0.054	0.015	30
Chica cavefish	0.047	0.012	30

1 Values are averages of three independent trials.
